# Immunoassays Based on *Penicillium marneffei* Mp1p Derived from *Pichia pastoris* Expression System for Diagnosis of Penicilliosis

**DOI:** 10.1371/journal.pone.0028796

**Published:** 2011-12-21

**Authors:** Yan-Fang Wang, Jian-Piao Cai, Ya-Di Wang, Hui Dong, Wei Hao, Ling-Xiao Jiang, Jun Long, Cheman Chan, Patrick C. Y. Woo, Susanna K. P. Lau, Kwok-Yung Yuen, Xiao-Yan Che

**Affiliations:** 1 Center for Clinical Laboratory, Zhujiang Hospital, Southern Medical University, Guangzhou, People's Republic of China; 2 Shanghai-MOST Key Laboratory for Disease and Health Genomics, Chinese National Human Genome Center at Shanghai, Shanghai, People's Republic of China; 3 Department of Microbiology, The University of Hong Kong, Hong Kong Special Administrative Region, People's Republic of China; Albert Einstein College of Medicine, United States of America

## Abstract

**Background:**

*Penicillium marneffei* is a dimorphic fungus endemic in Southeast Asia. It can cause fatal penicilliosis in humans, particularly in HIV-infected people. Diagnosis of this infection is difficult because its clinical manifestations are not distinctive. Specialized laboratory tests are necessary to establish a definitive diagnosis for successful management. We have demonstrated previously that a cell wall mannoprotein Mp1p, abundant in *P. marneffei,* is a potential biomarker for diagnosis of *P. marneffei* infections. In the present study, we describe immunoassays based on Mp1p derived from the yeast *Pichia pastoris* expression system.

**Methodology/Principal Findings:**

We generated monoclonal antibodies (MAbs) and rabbit polyclonal antibodies (PAbs) against Mp1p expressed in *P. pastoris*. Subsequently, we developed two Mp1p antigen capture ELISAs which employed MAbs for both the capture and detecting antibodies (MAb-MAb pair) or PAbs and MAbs as the capture and detecting antibodies (PAbs-MAb pair) respectively. The two Mp1p antigen ELISAs detected Mp1p specifically in cultures of *P. marneffei* yeast phase at 37–40°C and had no cross-reaction with other tested pathogenic fungi. The sensitivities and specificities of the two antigen assays were found to be 55% (11/20) and 99.6% (538/540) for MAb-MAb Mp1p ELISA, and 75% (15/20) and 99.4% (537/540) for PAbs-MAb Mp1p ELISA performed using 20 sera with culture-confirmed penicilliosis, and 540 control sera from 15 other mycosis patients and 525 healthy donors. Meanwhile, we also developed an anti-Mp1p IgG antibody ELISA with an evaluated sensitivity of 30% (6/20) and a specificity of 98.5% (532/540) using the same sera. Furthermore, combining the results of Mp1p antigen and antibody detection improved the sensitivity of diagnosis to 100% (20/20).

**Conclusions/Significance:**

Simultaneous detection of antigen and antibody using the immunoassays based on Mp1p derived from *P. pastoris* greatly improves detection sensitivity. The procedures should be useful for the routine diagnosis of penicilliosis.

## Introduction

*Penicillium marneffei* is an emerging fungus endemic in Southeast Asian regions, including Burma, Cambodia, Indonesia, Malaysia, Thailand, Vietnam, and southern parts of China [1], [2]. Penicilliosis, the systemic disease caused by *P. marneffei*, occurs in both immunocompetent and immunocompromised patients, particularly in AIDS patients. The infection mainly affects HIV-infected patients and is considered to be one of the most common opportunistic infections among AIDS patients in the endemic regions, and is therefore regarded as an indicator disease for AIDS [3], [4]. In addition, infections by *P. marneffei* have been reported in visitors who had travelled to the endemic regions [4].

Penicilliosis is a disseminated and progressive disease with high mortality. However, successful therapeutic management has been hampered by an absence of rapid and accurate diagnosis due to its non-specific symptoms and similar biological characteristics to other common pathogenic fungi, such as *Histoplasma capsulatum*, *Cryptococcus neoformans* and various *Aspergillus* species [5], [6]. A number of diagnostic methods based on antibody as well as antigen detection have been developed [7], [8], [9], [10], but they have not been used for routine diagnosis. The antibody tests were developed using crude extracts of fungal antigen and the inherent batch-to-batch variation makes these tests not amenable to standardization. Our aim was to develop the assays using antibodies against pure antigen that would show improved sensitivity and specificity. We have previously cloned a gene that encodes Mp1p, a cell wall antigenic mannoprotein that is abundant in both yeast and mycelia phases of *P. marneffei* [11]. By using the polyclonal antibodies (PAbs) raised against recombinant Mp1p (rMp1p) protein expressed in *E. coli*, we established an ELISA-based antibody and antigen assay for diagnosis of *P. marneffei* infections [12], [13]. To establish more sensitive diagnostic methods, in the present study we produced Mp1p protein using the *Pichia pastoris* expression system, which may provide the necessary post-translational modifications, such as glycosylation, to produce a protein that may be structurally closer to its native form [14]. Next, we generated both monoclonal antibodies (MAbs) and rabbit PAbs against *P. pastoris* derived Mp1p. We also developed two optimal Mp1p antigen capture ELISA assays which employed only MAbs, or PAbs and MAb as the capture and detecting antibodies respectively. Furthermore, we developed an ELISA-based antibody assay with rMp1p derived from *P. pastoris*. The feasibility of using simultaneous Mp1p antigen and antibody detection by the *P. pastoris* derived Mp1p based assays in diagnosis of penicilliosis is discussed.

## Materials and Methods

### Ethics Statement

The use of patient serum samples was approved by Institutional Review Board of the University of Hong Kong/Hospital Authority Hong Kong West Cluster under protocol no. UW 04-278 T/600 for all samples collected since September 2004. Control sera were collected specifically for this study. Informed written consent was obtained from each subject. The use of control sera obtained from blood donors and patient sera from an already-existing collection was approved by the Ethics Committee of the Zhujiang Hospital of Southern Medical University, Guangzhou, China, with the following reference number: ZJYY-2010-YXJYZX-001.

### Clinical specimens and Strains

Twenty serum specimens were obtained from 14 penicilliosis patients diagnosed by positive culture from blood or bone marrow aspirate specimens, of which 3 specimens from 2 patients were co-infected with *Cryptococcus neoformans*. Six of the 14 patients had 2 consecutive sera which were collected every three days. Sera were collected from patients with other fungal diseases, including seven with invasive aspergillosis, four with systemic candidiasis, two with pulmonary pseudallescheriasis and two with pulmonary aspergilloma. All patients with invasive aspergillosis had underlying haematological malignancy and multilobar pulmonary involvement with multiple positive smears and cultures from bronchoalveolar lavage fluid or sputum specimens. In addition, four cases were confirmed by histology from biopsy or postmortem examination. All patients with systemic candidiasis were neutropenic and had positive blood cultures. Pulmonary aspergilloma was diagnosed by positive culture from sputum or lung tissue and histopathology of resected lung tissue. The 525 negative control serum specimens were obtained from blood donors who were healthy at the time of participation.

A *P. marneffei* PM4 strain, *Aspergillus spp.* (*A. fumigatus*, *A. flavus*, *A. terreus*, and *A. niger*), and *C. neoformans* were obtained from the Department of Microbiology, University of Hong Kong. *Candida spp.* (*C. albicans*, *C. glabrata*, *C. tropicalis*, *C. krusei*, and *C. parapsilosis*) were obtained from the Research Centre for Medical Mycology, Beijing University, China [15].

The fungal culture filtrates from different growth conditions were prepared as described previously [11], [12], [15]. *P. marneffei* was first cultured on Sabouraud agar plates (Guangzhou Letongtai Biotech Co., Ltd, China) at 37°C or 28°C for 7 to 10 days. Culture supernatants were obtained by inoculating fungal cells from plates into RPMI-1640 (GIBCO, Carlsbad, CA). Cultures were shaken at 40°C, 37°C or 28°C for 5 to 6 days, centrifuged at 5000 rpm for 20 min, passed through a 0.45-µm-pore-size filter (Corning Inc., N.Y.) and stored at −80°C until used.

### Expression of rMp1p in *Pichia pastoris*

The Mp1p gene encoding a secreted cell wall mannoprotein in *P. marneffei* was characterized in a previous study [11]. To acquire a soluble protein, the N-terminal cleavable signal peptide and the C-terminal cleavable glycosylphosphatidylinositol (GPI) domain were removed from the full-length Mp1p gene and cloned into the *P. pastoris* expression vector pPIC9K (Invitrogen, Carlsbad, CA) [11]. The truncated Mp1p gene in *P. pastoris* strain GS115 (Invitrogen) was expressed and identified according to manufacturer's instructions. A large scale expression of recombinant Mp1p protein (rMp1p) was optimized and the protein was purified by Ni-nitrilotriacetic acid affinity chromatography (Qiagen, Hilden, Germany). The purity of rMp1p was assessed by sodium dodecyl sulfate-polyacrylamide gels (SDS-PAGE) and western blotting as described previously [15]. In brief, the purified rMp1p was separated electrophoretically in a 12.5% gel and transferred to a nitrocellulose membrane. After blocking with 3% BSA (Sigma-Aldrich), the membrane was incubated with anti-serum from guinea pigs immunized with purified rMp1p from *E.coli* [11] and anti-His monoclonal antibodies for 1 h at 37°C. After washing, the membrane was incubated with horseradish peroxidase (HRP; Sigma-Aldrich) conjugated goat anti-rabbit and goat anti-mouse antibody for 30 min at 37°C, and developed by incubation with Amersham ECL Advance Western Blotting Detection Kit (GE Healthcare, Fairfield, CT). The concentration of purified rMp1p was determined by using the Bicinchoninic Acid Protein Assay Kit (Sigma-Aldrich, St. Louis, MO) according to the manufacturer's instructions.

### Preparation of MAbs and PAbs against Mp1p

The process of preparation and identification of MAbs against Mp1p was described in our previous study [15], [16]. In brief, BALB/c mice were immunized intradermally with 50 µg of purified rMp1p emusified with Freund's adjuvant (Sigma-Aldrich) at ten-day intervals for 8 weeks followed by intravenously administered boosters for three days before fusion. Antibodies specific for *P. marneffei* in the hybridoma cell culture supernatant were screened by indirect ELISA using rMp1p and indirect immunofluorescent staining (IFA) using slides with immobilized fungal cells killed in 3.7% formalin solution overnight at 4°C. The isotypes of the MAbs were determined using a commercially available mouse MAb isotyping kit (Zymed Laboratories, Carlsbad, CA). MAbs were purified from ascitic fluid by ammonium sulfate precipitation and were conjugated with biotin using the EZ-Link® Sulfo-NHS-Biotinylation Kit following the manufacturer's instructions (Pierce Biotechnology, Rockford, IL). Rabbit polyclonal antibodies (PAbs) against rMp1p were produced in New Zealand White rabbits. Rabbits were immunized subcutaneously with 500 µg of purified rMp1p emulsified with Freund's adjuvant (Sigma-Aldrich) at ten-day intervals for 8 weeks followed by intravenous boosters for three days before collection of serum. The IgG fraction was purified by protein A column chromatography (GE Healthcare, Chalfont St. Giles, United Kingdom) and the purified MAbs and PAbs specific for *P. marneffei* were identified by IFA as described previously [15].

### Identification of MAb epitope groups by competitive ELISA

The epitopes groups of the MAbs were analyzed by competitive ELISA as described previously [16] with rMp1p as the coating antigen. Microwell plates (Costar, Corning, N.Y.) were coated with 100 µl/well of rMp1p at a concentration of 1 µg/ml in carbonate-bicarbonate (pH 9.6) coating buffer overnight at 4°C. Afterwards, the wells were blocked with 3% BSA in phosphate buffered saline for 2 h, and then a constant concentration of one of the non-biotinylated MAbs (50 µl) was incubated with an optimal concentration of different biotinylated MAbs (50 µl) for 1 h at 26°C. After the plates were washed, streptavidin-HRP (Sigma-Aldrich) was added and plates were incubated for 30 min at 26°C. The plates were washed, and the binding of the biotinylated MAb was detected by the addition of tetramethylbenzidine (TMB) substrate (KPL, Gaithersburg, MD). The reaction was stopped after 10 min by the addition of 1 N sulfuric acid, and the plates then were examined in an ELISA plate reader (Bio-Tek, Winooski, VT). An unlabeled MAb specific for dengue virus serotype 2 nonstructural protein 1 [16] was used as irrelevant control. The distinct binding epitopes of MAbs were determined as described previously [16].

### Development of Mp1p antigen capture ELISAs

The procedure for the optimal antigen capture ELISA was carried out as described previously with modifications [16]. In brief, microwell plates (Costar, Corning) were coated with 100 µl/well of capture antibodies (MAb or PAbs) overnight at 4°C followed by incubation with a blocking reagent containing 2.5 g Casein sodium salt, 1.21 g Tris-base, 2 g gelatin, 20 g sucrose, 0.2 g Merthiolate, and 5 ml Tween20 in 1000 ml dH2O (Sigma-Aldrich). After removal of the blocking solution, 100 µl of of culture filtrates of various fungal pathogens serially diluted or human sera diluted 1∶10 in 0.1% BSA were added per well and incubated at 37°C for 1 h. After the plates were washed, biotinylated MAb (100 µl/well) was added and incubated for 30 min at 26°C. Following incubation with streptavidin-HRP (Sigma-Aldrich), TMB substrate was added. The reaction was stopped after 10 min by the addition of 0.3 N sulfuric acid, and the plates were then examined in an ELISA plate reader (Bio-Tek) at 450 nm.

### Development of indirect Mp1p antibody ELISA

Microwell plates (Costar, Corning) were coated with 10 ng of rMp1p in a total volume of 100 µl/well overnight at 4°C followed by incubation with a blocking regent (the same as mentioned in Mp1p antigen capture ELISAs). After removal of the blocking solution, 100 µl/well of human sera diluted 1∶100 with 0.1% BSA were added, and incubated for 1 h at 37°C. After washing, 100 µl/well of goat anti-human IgG-HRP (Sigma-Aldrich) diluted 1∶8000 and TMB substrate was added. The reaction was stopped after 10 min by the addition of 0.3 N sulfuric acid and absorbance was determined as described above.

### Statistical analysis

The categorical variables between the two antigen ELISA methods and each combined antigen ELISA/anti-Mp1p IgG ELISA methods were compared using McNemar test.

## Results

### Expression of Mp1p in *P. pastoris* and production of Mp1p-specific MAbs and PAbs

**Figure 1:**
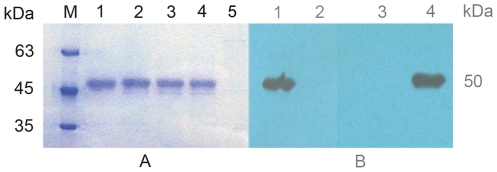
SDS-PAGE (A) and Western blotting (B) analysis of rMp1p expressed in *Pichia pastoris*. (A) M: protein molecular-mass marker; Lane 1: purified rMp1p; Lane 2 to 4: induced supernatant of positive transformant (pPIC9K-Mp1p/GS115) at 3, 4 and 5 days, respectively; Lane 5: induced supernatant of control transformant (pPIC9K/GS115); (B) Lane 1: the serum from a guinea pig immunized with rMp1p from *E.coli*; Lane 2: the serum from guinea pigs pre-immunization; Lane 3: irrelevant MAb control; Lane 4: anti-His MAb.

We have previously described the production of PAbs and MAbs against the rMp1p derived from *E. coli* [11], [17]. In this study, rMp1p was produced in a yeast protein expression system. To achieve protein secretion, a truncated form of the Mp1p gene, where the N-terminal cleavable signal peptide and C-terminal GPI membrane attachment signal sequence were removed [11], and then the Mp1p gene was cloned downstream of the α-factor secretion domain in the *P. pastoris* vector pPIC9K. As shown in **Figure 1**, rMp1p was abundantly secreted in culture supernatants, and the size was consistent with the expected size of 50 kDa (**Figure 1** **, A**). Western blotting analysis of rMp1p with serum from guinea pigs immunized with *E.coli* derived rMp1p [11] and anti-His MAb demonstrated the immunoreactivity of the rMp1p expressed from *P. pastoris* (**Figure 1** **, B**).

We prepared anti-rMp1p PAbs and 18 anti-rMp1p MAbs by using the rMp1p derived from *P. pastoris* to immunize rabbits and mice respectively. MAbs specific for Mp1p were selected according to a strong positive ELISA reaction with rMp1p and further confirmed by IFA with a strong positive reaction to *P. marneffei* cells in yeast phase. The specificities of the MAbs and PAbs were identified to be exclusively specific for *P. marneffei* cells in yeast phase with no reactivity to *P. marneffei* cells in mycelia phase and other fungi.

In order to develop a MAb-MAb ELISA to detect Mp1p, we need to identify two MAbs that bind to different epitopes. Competitive ELISA of all possible pairs of MAbs was employed to determine the epitope specificity of these MAbs using rMp1p as the coating antigen. While most of the MAbs recognized a common epitope (IV), at least four different epitopes on the Mp1p, were identified as determined by the inhibition of different MAbs (**Table 1**). The above results indicate that these MAbs or MAb/PAbs combinations can be useful for development of two-site sandwich antigen capture assays for detection of *P. marneffei*.

**Table 1:** The Characteristics of 18 anti-Mp1p MAbs.

MAb	Epitope^a^	Isotype	ELISA^b^	Reactivity with *P. marneffei* (IFA)	
				Yeast phase	Mycelia phase
M8	I	IgG	++	+	−
M4	II	IgG1	+++	+	−
M14	III	IgG1	++	+	−
M1	IV	IgG1	+++	+	−
M2	IV	IgG1	+++	+	−
M3	IV	IgG1	+++	+	−
M5	IV	IgG1	+++	+	−
M6	IV	IgG1	+++	+	−
M7	IV	IgG2a	+++	+	−
M9	IV	IgG2b	+++	+	−
M10	IV	IgG1	++	+	−
M11	IV	IgG2a	+++	+	−
M12	IV	IgG2b	+++	+	−
M13	IV	IgG1	++	+	−
M15	IV	IgG	+	+	−
M16	IV	IgG1	++	+	−
M17	IV	IgG2a	++	+	−
M18	IV	IgG2b	++	+	−

a The competition ELISA was performed to analyze the epitope groups of the different MAbs. Eighteen MAbs were found to fall into four groups, with each group reacting with the same epitope or sterical overlapping epitopes on Mp1p. The experiment was repeated with similar results.

b The purified rMp1p were used as the coating antigen and reacted with cell supernatants of hybridomas secreted anti-Mp1p MAbs. The absorbance was measured at 450 nm: +: OD_450_ = 1–1.5; ++: OD_450_ = 1.5–2; +++: OD_450_>2.

### Specificity and sensitivity of the Mp1p antigen ELISAs in detection of rMp1p and fungal culture filtrates

**Figure 2:**
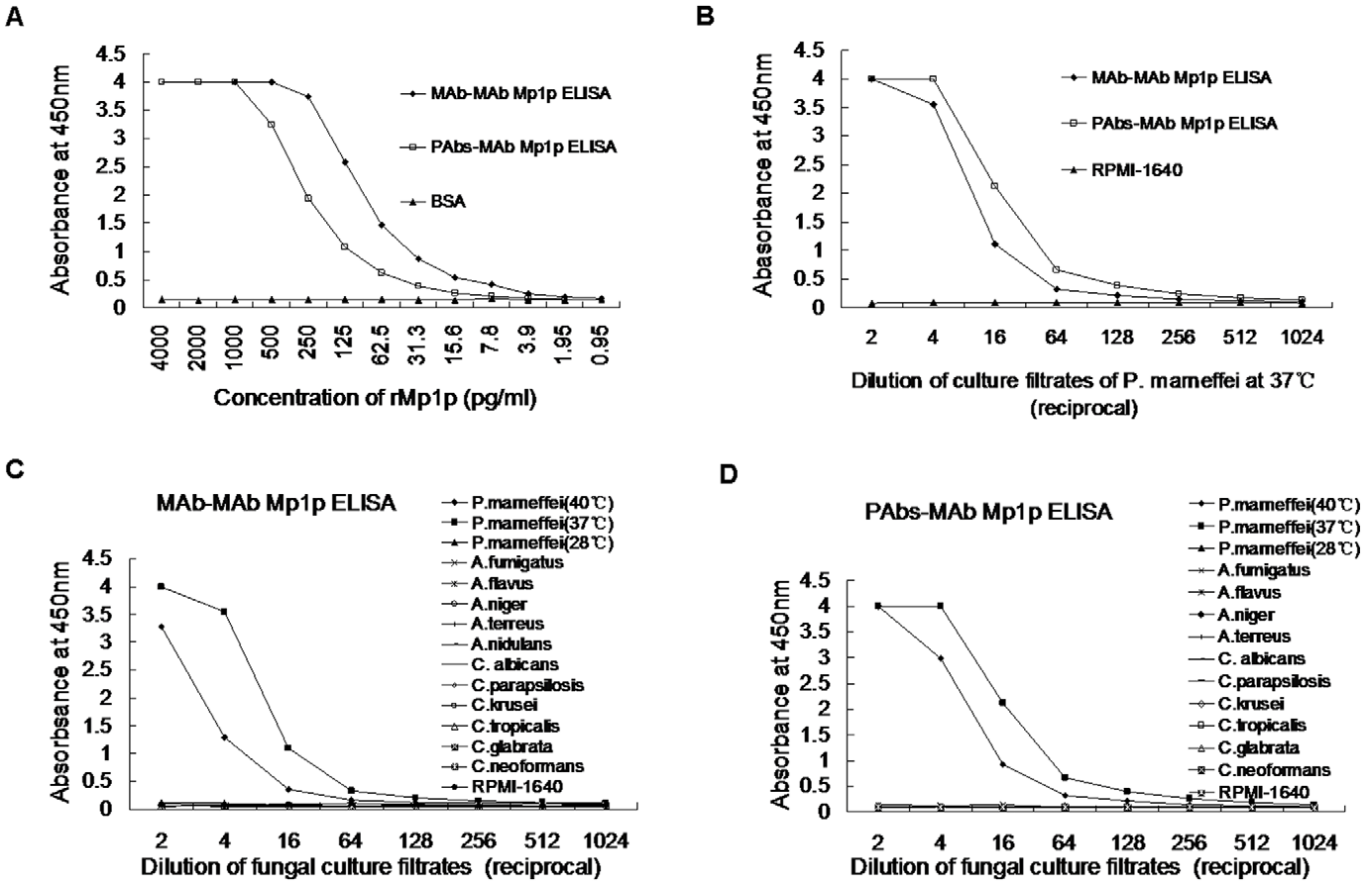
Evaluation of the sensitivity and specificity of two Mp1p antigen ELISAs. (A, B): evaluation of the sensitivity by detection of rMp1p and natural Mp1p in *P. marneffei* cells in yeast phase cultured at 37°C by two Mp1p antigen ELISAs; (C, D): evaluation of the specificity by detection of Mp1p in kinds of fungal cultured filtrates by MAb-MAb Mp1p ELISA (C) and PAbs-MAb Mp1p ELISA (D). The positive OD_450_ values are restricted to cultured filtrate of *P. marneffei* in yeast phase, and are higher when cultured at 37°C.

To establish MAb-MAb ELISA or PAbs-MAb ELISA for detection of Mp1p in *P. marneffei*, all possible pairs of MAbs or MAb/PAbs were tested to select the optimal capturer-detector, based on the sensitivity in detection of both rMp1p and native Mp1p in culture filtrates of *P. marneffei* in yeast phase. From these studies MAb M4 and M12 (**Table 1**) were selected as capture and detecting antibody for the MAb-MAb pair, and PAbs with M8 for the PAbs-MAb pair. The MAb-MAb Mp1p ELISA was more sensitive than the PAbs-MAb Mp1p ELISA with a detection limit of 7.8 pg/ml rMp1p, while the lower limit of the PAbs-MAb Mp1p ELISA was approximately 62.5 pg/ml. However, the MAb-MAb Mp1p ELISA was less sensitive than PAbs-MAb Mp1p ELISA when culture filtrates from the yeast phase of *P. marneffei* cells were used (**Figure 2** **, A; B**).

Next, we further analyzed for cross-reactivity of the two Mp1p ELISAs with other related and opportunistic fungi. Only filtrates of identically inoculated *P. marneffei* yeast phase cell cultures at 37°C or 40°C gave positive signals (**Figure 2** **, C; D**). In addition, the culture at 37°C had higher OD_450_ values than those cultured at 40°C, which may due to more Mp1p being secreted at normal body temperature (37°C) than during fever (40°C) or different modifications of Mp1p at different temperature. It is interesting that both Mp1p antigen assays failed to detect Mp1p in cultures of mycelia phase at 28°C. Finally, none of the other fungal cultures gave positive results, demonstrating that the two Mp1p assays were specific for the detection of *P. marneffei* cells in yeast phase, the only form existing in humans, with no cross-reaction to the other tested common pathogenic fungi.

### Evaluation of the two Mp1p antigen ELISAs and anti-Mp1p antibody ELISA in positive detection of penicilliosis patient sera

Our previous study [12] showed the improved sensitivity of combination detection strategies involving Mp1p and Mp1p-specific antibody. This encouraged us to apply a similar protocol using the anti-Mp1p IgG antibody ELISA with rMp1p derived from *P. pastoris*. The baseline of the two Mp1p antigen ELISAs and of the anti-Mp1p IgG ELISA was determined by using serum samples from 525 healthy donors. All serum samples were analyzed by the two Mp1p antigen ELISAs at 1∶10 dilution and by the anti-Mp1p IgG ELISA at 1∶100 dilution. The cutoff values were determined as the mean OD_450_ value of negative controls plus 5-times the standard deviation [16], yielding values of 0.317, 0.208, and 0.511 for the MAb-MAb, PAbs-MAb Mp1p ELISAs, and anti-Mp1p IgG ELISA respectively. If a sample yielded an OD_450_ value above the cutoff value of these assays, the result was considered positive. Next, a panel of patient sera was used to evaluate these assays. Of 20 sera obtained from 14 penicilliosis patients, in which 3 specimens from 2 patients were co-infected with *C. neoformans*, 11 (55%) and 15 (75%) sera at 1∶10 dilution were positive for Mp1p antigen as detected by MAb-MAb Mp1p ELISA and PAbs-MAb Mp1p ELISA, respectively, while 6 (30%) sera at 1∶100 dilution were positive for Mp1p-specific IgG antibody ELISA. It is interesting that the two samples from patient 4 were obtained before and after treatment with anti-fungal drug, and the results showed a large decrease in antigen, showing the efficacy of the treatment. Although the IgG ELISA alone has almost no diagnostic value, the results in 19 of 20 penicilliosis patient sera are complementary to those from the antigen tests, suggesting that the antibodies are successful in sequestering the antigen.

Fifteen serum samples from other mycosis patients were tested for Mp1p antigen, with only one pseudallescheriasis patient sample yielding a weak positive by PAbs-MAb Mp1p ELISA. Interestingly, of these 15 non-penicilliosis patients, 5 (33.3%) patients (2 with aspergillosis and 3 with candidiasis) were positive for Mp1p-specific IgG by anti-Mp1p IgG ELISA. The detailed test information regarding these patients is summarized in **Table 2**. Although patients 13 and 14 were co-infected with *C. neoformans*, the positive results obtained for these patients were likely due to penicilliosis since *C. neoformans* extracts *in vitro* were shown to be negative in **Figure 2**. Again, the IgG ELISA alone has little diagnostic value with 6/15 false positive. In this case it seems likely that the IgG is recognizing an antigen common to several fungal pathogens, although an authentic antibody response due to environmental exposure cannot be ruled out.

**Table 2:** Results of Mp1p antigen and Mp1p specific IgG in patient sera by two Mp1p antigen ELISAs and an Mp1p antibody ELISA.

Patient No./Diagnosis	OD_450_ values and results (+/−)		
	MAb-MAb Mp1p ELISA	PAbs-MAb Mp1p ELISA	Mp1p IgG ELISA
1/Penicilliosis	0.102 (−)	0.099 (−)	0.818 (+)
2/Penicilliosis	0.095 (−)	0.140 (−)	1.998 (+)
	0.100 (−)	0.109 (−)	1.524 (+)
3/Penicilliosis	0.899 (+)	4.000 (+)	0.078 (−)
	2.087 (+)	3.889 (+)	0.084 (−)
4/Penicilliosis	0.557 (+)	1.935 (+)	0.796 (+)
	0.261 (−)	0.447 (+)	0.342 (−)
5/Penicilliosis	0.114 (−)	0.643 (+)	0.089 (−)
6/Penicilliosis	0.105 (−)	0.138 (−)	1.891 (+)
7/Penicilliosis	3.991 (+)	4.000 (+)	0.084 (−)
8/Penicilliosis	0.108 (−)	0.321 (+)	0.068 (−)
	0.138 (−)	0.315 (+)	0.115 (−)
9/Penicilliosis	3.989 (+)	4.000 (+)	0.163 (−)
10/Penicilliosis	0.102 (−)	0.099 (−)	1.285 (+)
11/Penicilliosis	4.000 (+)	4.000 (+)	0.108 (−)
	4.000 (+)	4.000 (+)	0.074 (−)
12/Penicilliosis	1.275 (+)	4.000 (+)	0.134 (−)
13/Penicilliosis^a^	0.832 (+)	3.103 (+)	0.061 (−)
14/Penicilliosis^a^	4.000 (+)	4.000 (+)	0.094 (−)
	3.995 (+)	4.000 (+)	0.098 (−)
15/Invasive aspergillosis	0.110 (−)	0.141 (−)	0.096 (−)
16/Invasive aspergillosis	0.079 (−)	0.109 (−)	0.095 (−)
17/Invasive aspergillosis	0.112 (−)	0.112 (−)	0.099 (−)
18/Invasive aspergillosis	0.109 (−)	0.098 (−)	0.193 (−)
19/Invasive aspergillosis	0.130 (−)	0.104 (−)	1.048 (+)
20/Invasive aspergillosis	0.094 (−)	0.090 (−)	0.167 (−)
21/Invasive aspergillosis	0.099 (−)	0.094 (−)	0.052 (−)
22/COPD/aspergillosis	0.089 (−)	0.121 (−)	1.111 (+)
23/Bronchiectasis/aspergillosis	0.092 (−)	0.120 (−)	0.325 (−)
24/Invasive candidiasis	0.102 (−)	0.102 (−)	1.748 (+)
25/Invasive candidiasis	0.096 (−)	0.098 (−)	0.115 (−)
26/Invasive candidiasis	0.095 (−)	0.101 (−)	0.795 (+)
27/Invasive candidiasis	0.108 (−)	0.101 (−)	0.739 (+)
28/Pseudallescheriasis	0.094 (−)	0.176 (−)	0.163 (−)
29/Pseudallescheriasis	0.096 (−)	0.228 (+)	0.060 (−)

a penicilliosis patients co-infected with *C. neoformans*.

The overall diagnostic accuracy of Mp1p antigen and antibody ELISA for the identification of penicilliosis is summarized in **Table 3**. The specificities of these assays were 99.6% (538/540), 99.4% (537/540), and 98.5% (532/540) for MAb-MAb Mp1p ELISA, PAbs-MAb Mp1p ELISA, and anti-Mp1p IgG ELISA, respectively, as determined using 540 controls including 15 patients with non-penicilliosis mycoses and 525 healthy volunteers. When a combined detection strategy using both Mp1p antigen- and antibody-based ELISA was used for the diagnosis of penicilliosis, the sensitivity, specificity, positive predictive value (PPV), and negative predictive value (NPV) were 85.0%, 98.1%, 63.0%, and 99.4% for MAb-MAb Mp1p/anti-Mp1p IgG ELISA, and 100%, 98.0%, 64.5%, and 100% for PAbs-MAb Mp1p/anti-Mp1p IgG ELISA, respectively.

**Table 3:** Performance of Mp1p antigen and antibody ELISAs for diagnosis of Penicilliosis.

Mp1p antigen and antibody assays^a^	Clinical samples, No. of positive/No. of sera (%)			Overall diagnostic characteristic (%, n = 560), (95% CI)			
	Penicilliosis (n = 20)	Other fungal diseases (n = 15)	Normal sera (n = 525)	Sensitivity	Specificity	PPV	NPV
1. MAb-MAb Mp1p ELISA	11/20 (55.0)	0/15 (0.0)	2/525 (0.4)	55.0 (31.0–77.0)	99.6 (99.1–100)	84.6 (55.0–98.0)	98.4 (97.3–99.5)
2. PAbs-MAb Mp1p ELISA	15/20 (75.0)	1/15 (6.7)	2/525 (0.4)	75.0 (51.0–91.0)	99.4 (98.7–100)	83.3 (59.0–96.0)	99.1 (98.3–99.9)
3. Mp1p antibody ELISA	6/20 (30.0)	5/15 (33.3)	3/525 (0.6)	30.0 (12.0–54.0)	98.5 (97.5–99.5)	42.9 (18.0–71.0)	97.4 (96.1–98.7)
4. MAb-MAb Mp1p ELISA Plus Mp1p antibody ELISA	17/20 (85.0)	5/15 (33.3)	5/525 (1.0)	85.0 (62.0–97.0)	98.1 (97.0–99.2)	63.0 (42.0–81.0)	99.4 (99.0–99.7)
5. PAbs-MAb Mp1p ELISA plus Mp1p antibody ELISA	20/20 (100)	6/15 (40.0)	5/525 (1.0)	100 (83.0–100)	98.0 (96.8–99.2)	64.5 (49.0–83.0)	100 (97.5–100)

a There was no significant difference (McNemar test, *P*>0.1, n = 560) comparing assay 1 *versus* 2, or 4 *versus* 5. There were very significant differences (McNemar test, *P*<0.005, n = 560) comparing assay 1 *versus* 4, and 2 *versus* 5.

## Discussion

We have demonstrated previously that a cell wall mannoprotein, Mp1p, abundant in *P. marneffei,* is a potential biomarker for serodiagnosis of active *P. marneffei* infections [11], [12], [13]. This report describes the production of a panel of MAbs and PAbs against *P. pastoris*-derived Mp1p that were used to develop two Mp1p antigen capture ELISAs, one by using MAbs as both the capture and detecting antibodies, and the other by using PAbs and MAb as the respective capture and detecting antibodies. The MAb-MAb Mp1p ELISA displayed an 8-fold higher rate of detection compared to that of the PAbs-MAb Mp1p ELISA when rMp1p was used as the target, but a 2-fold drop in sensitivity compared to the PAbs-MAb Mp1p ELISA when applied to culture filtrates. This may be due to differences in epitope exposure between *P. pastoris-*expressed rMp1p and native Mp1p in culture filtrates, or variation in Mp1p antigenicity between different *P. marneffei* isolates. Furthermore, the ELISA results using patient sera showed that the PAbs-MAb Mp1p ELISA was more sensitive (75%) than MAb-MAb Mp1p ELISA (55%), indicating that PAbs, when used as the capture antibody, may widen the spectrum of natural epitopes, found within fungal proteins.

Mp1p is a highly antigenic cell wall mannoprotein that can elicit an antibody response in infected patients [11], [12], [13]. Previous studies investigating the detection of anti-Mp1p antibodies by the *E. coli* BL21 derived rMp1p-based ELISA yielded a sensitivity of 80% and a specificity of 100% in penicilliosis patients [13], providing a proof-of-concept for the serodiagnostic value of Mp1p. In this study, we found that only 30% of penicilliosis patients were positive for anti-Mp1p antibodies by the *P. pastoris* expressed rMp1p based ELISA. This phenomenon of differential antibody response in penicilliosis patients is probably related to patients with different degrees of immunosuppression, who may produce low or undetectable levels of antibodies [13], [18]. It would have been interesting to test our assays on the same samples that were used previously to better characterize the improved value of the method. Unfortunately, those original samples are no longer available. We also found that there were cross-reactive anti-Mp1p antibodies in aspergillosis and candidiasis patients. Possibly, these patients had been infected with *P. marneffei* previously. Another explanation is that rMp1p derived from recombinant *P. pastoris,* may be highly mannosylated, and have cross-reactive epitopes with other fungal proteins. However, in this study, the antibodies raised against rMp1p recognized *P. marneffei* epitopes exclusively, as demonstrated both by ELISA and by IFA. The epitope-specific MAbs-based Mp1p antigen assay therefore displayed a high specificity in penicilliosis patients. A weak positive result was obtained with one sample from a patient with pseudallescheriasis by PAbs-MAb Mp1p ELISA, which suggested that cross-reactivity may exist between the Mp1p of *P. marneffei* and *Pseudallescheria boydii* or other *Scedosporium spp.*, such as *S. apiospermum* and *S. prolificans*. Future studies may be performed to test for potential cross-reactivity using more pathogenic fungal species such as *P. boydii.* When the PAbs raised against *P. pastoris* derived rMp1p were used as the capture antibodies and MAb as a detector to form the two-site sandwich antigen capture assay, the assay appears to have a higher sensitivity (75%) than previous Mp1p antigen assays that made use of two different PAbs raised against *E. coli* derived rMp1p (sensitivity of 65.4%) [12]. Strikingly, combining the results of the yeast *P. pastoris* derived Mp1p-based ELISA assays, we found that in 95% (19/20) of the penicilliosis specimens, the antigen test and the antibody test results are complementary to each other, with only 1 of 20 serum specimens having concomitantly significant Mp1p antigen and Mp1p antibodies. The value of complementation of results targeting the Mp1p antigen and anti-Mp1p antibodies was shown by the fact that when the results of both tests were combined, the diagnostic sensitivity increased to 100% in penicilliosis patients. However, evaluation of the actual sensitivity and specificity of the assays in prospective studies with a larger number of specimens from patients with *P. marneffei* infection and other fungal diseases is necessary.

In conclusion, this study describes the yeast *P. pastoris* derived *P. marneffei* Mp1p-based antigen and antibody tests for diagnosis of penicilliosis patients. The combined performances of Mp1p antigen and antibody testing facilitate enhanced diagnosis rates in *P. marneffei* infections. The procedures should be useful for the routine diagnosis of penicilliosis.
